# Diagnosis of Cattle Diseases Endemic to Sub-Saharan Africa: Evaluating a Low Cost Decision Support Tool in Use by Veterinary Personnel

**DOI:** 10.1371/journal.pone.0040687

**Published:** 2012-07-12

**Authors:** Mark C. Eisler, Joseph W. Magona, Crawford W. Revie

**Affiliations:** 1 School of Veterinary Sciences, Faculty of Medicine and Veterinary Medicine, University of Bristol, Bristol, United Kingdom; 2 Bulindi Zonal Agricultural Research and Development, Hoima, Uganda; 3 Atlantic Veterinary College, University of PEI, Charlottetown, Canada; Auburn University, United States of America

## Abstract

**Background:**

Diagnosis is key to control and prevention of livestock diseases. In areas of sub-Saharan Africa where private practitioners rarely replace Government veterinary services reduced in effectiveness by structural adjustment programmes, those who remain lack resources for diagnosis and might benefit from decision support.

**Methodology/Principal Findings:**

We evaluated whether a low-cost diagnostic decision support tool would lead to changes in clinical diagnostic practice by fifteen veterinary and animal health officers undertaking primary animal healthcare in Uganda. The eight diseases covered by the tool included 98% of all bovine diagnoses made before or after its introduction. It may therefore inform proportional morbidity in the area; breed, age and geographic location effects were consistent with current epidemiological understanding. Trypanosomosis, theileriosis, anaplasmosis, and parasitic gastroenteritis were the most common conditions among 713 bovine clinical cases diagnosed prior to introduction of the tool. Thereafter, in 747 bovine clinical cases estimated proportional morbidity of fasciolosis doubled, while theileriosis and parasitic gastroenteritis were diagnosed less commonly and the average number of clinical signs increased from 3.5 to 4.9 per case, with 28% of cases reporting six or more signs compared to 3% beforehand. Anaemia/pallor, weakness and staring coat contributed most to this increase, approximately doubling in number and were recorded in over half of all cases. Finally, although lack of a gold standard hindered objective assessment of whether the tool improved the reliability of diagnosis, informative concordance and misclassification matrices yielded useful insights into its role in the diagnostic process.

**Conclusions/Significance:**

The diagnostic decision support tool covered the majority of diagnoses made before or after its introduction, leading to a significant increase in the number of clinical signs recorded, suggesting this as a key beneficial consequence of its use. It may also inform approximate proportional morbidity and represent a useful epidemiological tool in poorly resourced areas.

## Introduction

Improved diagnosis is a prerequisite for effective management of endemic cattle diseases in sub-Saharan Africa. However this is currently constrained by the limited availability of suitably trained professional staff, field-level diagnostic tests and a general lack of knowledge about disease among livestock owners [1]. Moreover, under field conditions clinical diagnosis of these diseases is complicated by the occurrence of a combination of intestinal and haemoparasites, which mutually exacerbate each other’s pathogenic effects [2], [3]. Where multiple similar diseases occur decision support tools might facilitate differential diagnosis [4]. Current thinking in terms of veterinary service provision favours pen-side diagnostic tests and decision support technology suitable for use by farmers, extension workers and agro-veterinary traders; i.e. those who most often make the diagnosis and treatment decisions in rural African settings [1]. Recently a low cost decision support tool has been developed to aid the diagnosis of anaplasmosis, babesiosis, cowdriosis, fasciolosis, parasitic gastroenteritis, schistosomosis, theileriosis, and trypanosomosis in sub-Saharan Africa [5].

In this paper we describe the outcome of a study conducted to evaluate the effectiveness of the decision support tool as a diagnostic aid under field conditions by observing whether its introduction to veterinary and animal health officers undertaking primary animal health care in Uganda would lead to changes in clinical practice.

## Materials and Methods

### Diagnostic Decision Support Tool

**Figure 1:**
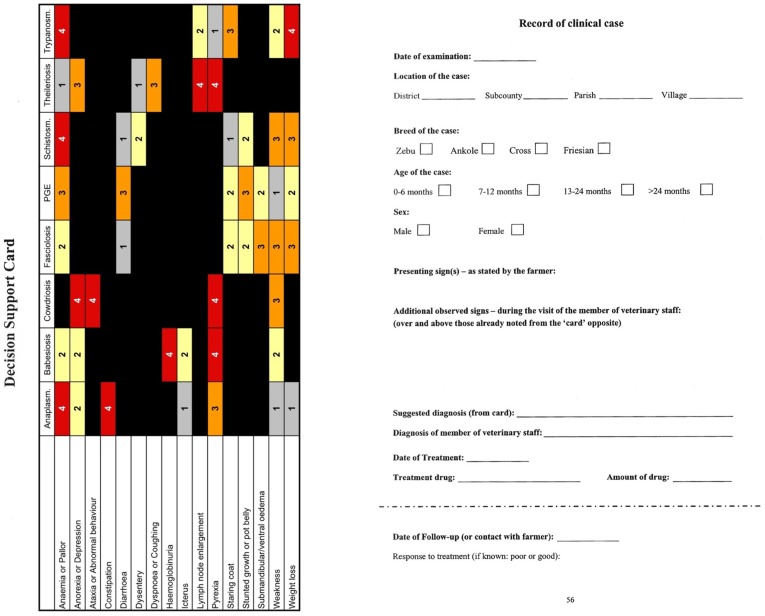
Template casebook used by participating Ugandan veterinary and animal health officers to record data in Phase-2 of the study. The template casebook was provided in the form of a printed notebook with the decision support tool appearing on each left hand page in landscape orientation so that clinical signs observed could be marked directly on it.

The low cost decision support tool developed to aid the diagnosis of endemic bovine infectious diseases in the mixed crop–livestock production system of sub-Saharan Africa takes the form of a simple printed card that relates each of the eight diseases considered to a number of clinical signs (Figure 1). The card depicts a grid comprising eight columns, one for each disease and sixteen rows, one for each clinical sign. The cells of the grid contain the numbers 1, 2, 3 or 4, these being weightings ascribed to each sign in terms of its importance for diagnosing each disease, or are blank (representing zero weighting). Weighting values were obtained by a Delphi survey of expert opinion [5]. The numbers are accentuated by appearing on a coloured background, red for the highest weighting value of 4, orange for the next highest value, 3, yellow for 2 and grey for the lowest weighting value of 1, thereby mitigating against the likelihood of a misread weighting value. In use, the clinician sums the values in each column including only weightings in rows representing observed clinical signs; the disease associated with the column giving the highest total is considered the most likely diagnosis. In this study, the card was implemented on A5-size paper using an ordinary colour printer and laminated in sealed plastic pouches to ensure durability and re-usability under field conditions. A version of the card was also produced within the case-books designed for data recording during the study as discussed below and illustrated in Figure 1.

### Study Area

**Figure 2:**
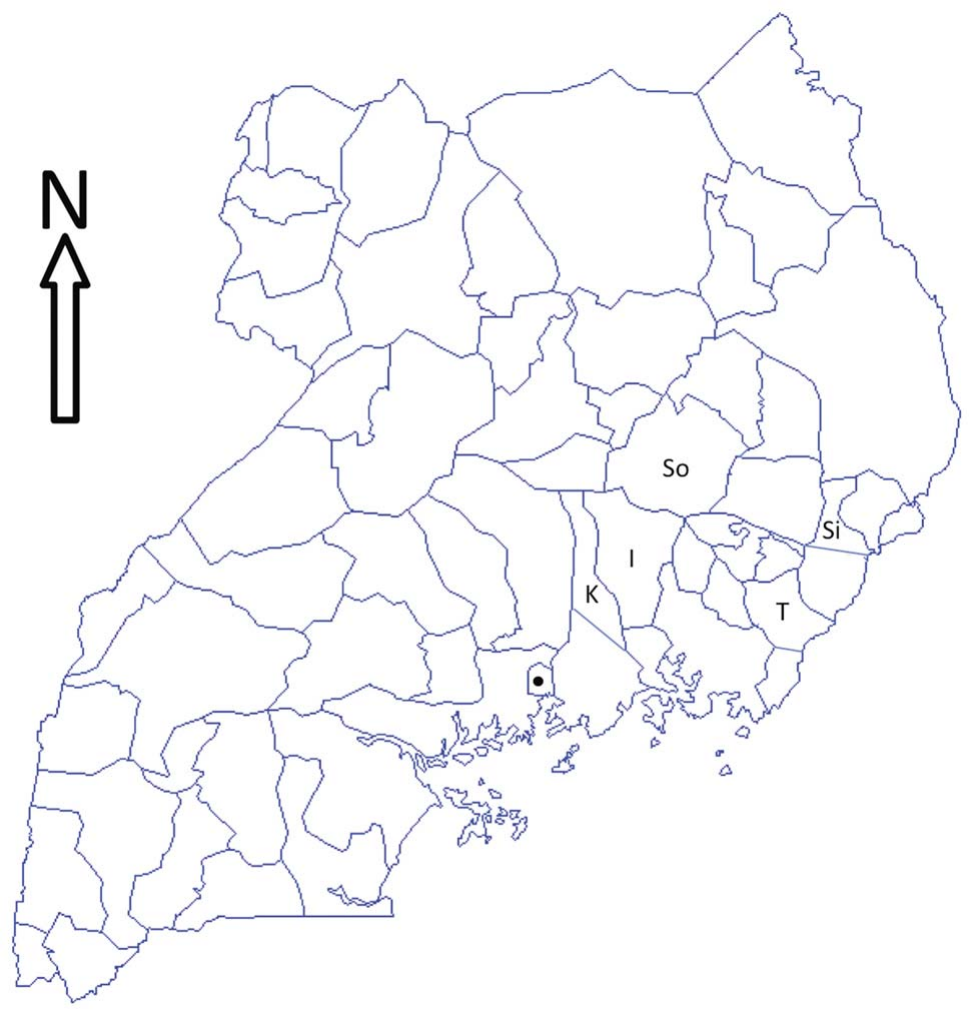
Map of Uganda showing the five districts in which the study was carried out. The study was carried out in Iganga (I), Kayunga (K), Sironko (Si), Soroti and Tororo (T) Districts in the eastern region of Uganda between January and May of 2005. Solid symbol is the Capital city, Kampala.

The study was carried out between January and May 2005 in Iganga, Kayunga, Sironko, Soroti and Tororo Districts in the eastern region of Uganda (Figure 2). Savannah grassland is the main vegetation in the study area, which receives 1200–1500 mm of rainfall annually distributed in a bimodal manner. Interspersed between two dry seasons are two wet seasons, March to May and September to November. The overall daily mean minimum temperature is 15°C and the mean maximum 27°C. Small seasonal variations in rainfall and temperatures exist among the districts included in the study [6].

The economically important, endemic, vector-borne and parasitic diseases of cattle in this area are typical for the East African region and include: anaplasmosis caused by *Anaplasma marginale* [7], [8], [9]; babesiosis caused by *Babesia bigemina* [10], [7], [9]; cowdriosis or heartwater, caused by *Ehrlichia ruminantium*, previously called *Cowdria ruminantium*, [11], [12]; fasciolosis caused by *Fasciola gigantica* [13], [14]; parasitic gastroenteritis caused most severely by *Haemonchus* sp. [8], [15]; schistosomiasis [16], [17]; theileriosis or East Coast fever caused by *Theileria parva*, [18], [19], [7], [8], [9]; and trypanosomosis caused by *Trypanosoma brucei*, *Trypanosoma congolense* and *Trypanosoma vivax* [20], [8], [21].

The elevation of the study area varies from lowland swamps and marshland, which are more widespread in Kayunga, Iganga and Soroti Districts and suitable for the snail intermediate hosts of fasciolosis and schistosomiasis, to higher altitudes on the slopes of Mt Elgon in Sironko District, where levels of infestation with the tick and tsetse vectors of anaplasmosis, babesiosis, cowdriosis, theileriosis and trypanosomosis may differ from those elsewhere [7], [22].

### Participants and Study Design

Fifteen clinical participants undertaking primary animal health care in each of the five districts were recruited to take part in the study, including District Veterinary Officers (DVOs), Veterinary Officers and Animal Health Officers. The DVO for each district was informed that the study would involve recording of clinical signs and case data from field visits, and requested to include participants at each of these three levels of qualification. However, the final selection of participants was at the discretion of each DVO.

The study was conducted in two phases. During Phase-1 case data from five districts across Uganda were recorded in the field using a standardised form. Each participant was asked to record as many cases as possible over a two-month period while undertaking normal clinical duties. Participants were informed that a minimum of 45 cases would be required to ensure their involvement in the second phase of the study. Details associated with each case were recorded in a standard format using the right-hand portion of the layout shown in Figure 1. Phase-1 took place between January and March.

After Phase-1 had been completed all 15 participants attended a workshop at the Livestock Health Research Institute (LIRI) in Tororo, Uganda, at which the participants were introduced to the decision support tool (DST); its potential use as a diagnostic aid was explained and some practice sessions illustrating its use in the field were conducted by the authors.

This workshop was followed by Phase-2 of the study during which each participant was once again asked to record data from at least 45 cases over a two-month period. However, during this phase the participants utilised the DST as part of their routine clinical examination and recorded data using a standardised form similar to that used in the earlier phase (i.e. the full format of the case-book shown in Figure 1). Phase-2 was conducted between April and June across the same five districts.

#### Study approval

The study protocol was assessed and approved by the Uganda National Council for Science and Technology.

#### Data storage and analysis

Basic data recorded on the forms used in both Phase-1 and Phase-2 included: date of examination; location of the case; breed, sex and age of the case; presenting signs as noted by the farmer; clinical signs observed by the participant; tentative diagnosis; date of treatment, with type and amount of drug used. For most cases follow-up visits were made, at which point the date and the participant's assessment of the response to treatment were recorded. The additional information recorded during Phase-2 related to the diagnosis suggested by the DST as being the most likely. In both the case of diagnoses made by the participant and those suggested by the DST it was possible for more than one tentative diagnosis to be specified. This typically indicated either the potential of concurrent disease or that the information available at time of diagnosis was not sufficient to specify a unique diagnosis.

At the end of each phase case data were obtained from all participants and entered into an electronic format. The data were originally stored in worksheets within Microsoft Excel but were subsequently uploaded to a Microsoft Access database to allow for the more complex querying and summary required to create appropriate data structures for analytical tasks. Summary statistics were produced using Microsoft Excel, while database queries were created in Microsoft Access.

#### DST diagnosis calculation

To expedite analysis and remove a potential source of error, DST diagnoses were calculated from participants’ clinical sign data using matrix algebra; the 8-by-16 DST matrix (Dx) was premultiplied by the transpose of an n-by-16 signs matrix (S), comprising n cases as rows and clinical signs coded 1 or 0 for presence or absence as columns ordered as on the DST, to yield an n-by-8 scores matrix (C):
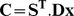



The scores matrix was then modified to represent diagnoses using an algorithm such that the maximum value(s) in each row was replaced by a weighting value (*w*), and all other values by zero; where ties occurred (i.e. the DST suggested more than one diagnosis), the usual weighting value (typically 12 to allow for 4 ties) was divided by the number of ties ensuring that the sum of weights for each row remained constant. Where none of the clinical signs on the DST were present, i.e. participants reported only ‘other’ signs, the full weighting value was recorded in an additional 9^th^ column of the modified scores matrix representing an ‘other’ category that otherwise contained zeros.

#### Proportional morbidity

An n-by-9 matrix of participants’ diagnoses (V) was constructed similarly with weights divided for multiple diagnoses as before, and diagnoses not listed on the DST in the ninth column. Proportional morbidity (P) was calculated for each of the 8 diagnoses listed on the DST plus the ninth ‘other’ category, as the proportion that diagnosis represented of all diagnoses by summation of the weights in each column of the n-by-9 matrix of DST or participants’ diagnoses, and further dividing each sum by the grand total of all weights (n x *w), i.e.*

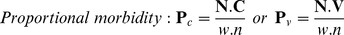
where *w* is the scalar weighting value, *n* is the number of cases and N is a row vector of 1s of length *n*.

#### Concordance

A matrix κ representing concordance of the DST diagnoses with the participants’ scores was calculated as follows:
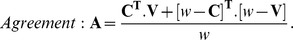


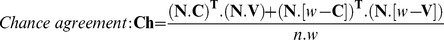



*Maximum agreement*: 
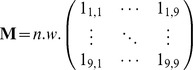




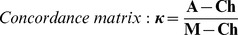
where ^T^ signifies the transpose of a matrix and the concordance matrix calculation uses element by corresponding element division of numerator by denominator 9-by-9 matrices.

Overall concordance (κ) for all nine diagnoses was calculated by summating the elements of the main diagonal, i.e. the trace (*tr)*, of the numerator and denominator prior to division:

*Overall concordance: 
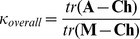
*

Nomenclature when describing the relative strength of agreement associated with kappa statistics followed published recommendations [23]: <0.000, poor; 0.000–0.200, slight; 0.201–0.400, fair; 0.401–0.600, moderate; 0.601–0.800, substantial; 0.801–1.000, almost perfect.

#### Misclassification

A simple misclassification matrix (M) for ‘positive’ agreement, uncorrected for chance, and scaled to show either participants’ diagnoses in rows as a proportion of each DST diagnosis in columns (M*_c_*) or DST diagnoses in rows as a proportion of each participants’ diagnosis in columns (M*_v_*), was calculated as:
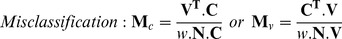
where each element of the numerator 9-by-9 matrix is divided by the value of the corresponding column of the denominator 1-by-9 row vector.

Differences in sign reporting and case outcomes between Phase-1 and Phase-2 were investigated using Chi-squared tests. All statistical analyses were carried out using Microsoft Excel, and MiniTab Version 14. Matrix algebra, algorithms and concordance (κ) were calculated using R version 2.14.2.

## Results

### Characteristics of Bovine Cases Reported by Participants

Before considering specific diseases or signs and any impact that the DST may or may not have had on diagnostic practice, it is useful to consider the breakdown of cases that were reported by the participants during the two phases of the study.

The participants reported on 713 and 751 bovine clinical cases in Phase-1 and Phase-2 respectively. The breakdown of these 1464 cases according to a number of key variables is shown in Table 1. This indicates that the composition of animals in terms of these variables remained broadly similar between the two phases. In both phases of the study, clinical examinations were conducted most often on cattle over 24-months of age, which were examined around three times more frequently than young cattle (0–6 months), with cattle in the intermediate age categories (7–12 and 12–24 months) falling between these two extremes.

**Table 1:** Breakdown of all cases (n = 1464) by key variables over the two phases of the study, pre and post-introduction of the DST.

	Phase	
	1	2
**Age group**		
0–6 months	13%	14%
7–12 months	25%	27%
13–24 months	22%	23%
>24 months	40%	36%
**Gender**		
Female	68%	62%
Male	32%	38%
**District**		
Iganga	17%	19%
Kayunga	19%	19%
Sironko	21%	20%
Soroti	21%	20%
Tororo	22%	22%
**Participant Type**		
Animal Health Officer	44%	47%
Veterinary Officer	28%	27%
District Veterinary Officer	28%	26%

Around twice as many female cattle were presented as were male; while Zebu cattle represented the most common breed, around half as many were identified as crossbred and much smaller proportions as Ankole and Friesian. All of the five districts in which data were recorded are roughly equally represented though the fact that all three participants from Kayunga were animal health officers rather than veterinary officers, resulted in this participant type being over-represented in the study as a whole.

### Clinical Signs Recorded by Participants

There were a total of 6178 clinical signs recorded for the 1460 cases investigated by the participants over the two phases of the study. The most commonly occurring sign was anorexia or depression, seen in over 55% of cases. Weight loss, staring coat, and fever were observed in almost half of all cases, while weakness, enlarged lymph nodes, and anaemia were also present in around 40% of cases. Diarrhoea and dyspnoea or coughing were seen in around a quarter of cases, with constipation and stunted growth or a pot belly being seen in just under 20% and 15% of cases respectively. The remaining signs were observed relatively infrequently (i.e. in around 5% or fewer cases).

Of interest is whether and how the reporting of signs changed over the course of the two phases in the study. Table 2 lists signs in order of the proportional change in their observation frequency between Phase-1 and Phase-2. An increase was seen in 13 out of the 16 clinical signs, which was significant (p<0.05) for 8 signs (Table 2); significant increases were observed for anaemia or pallor, weakness, starring coat and lymph node enlargement (p<0.001); for submandibular or ventral oedema, stunted or pot belly and weight loss (p<0.01); and for haemoglobinuria (p<0.05). There was an almost four-fold increase in the percentage of cases in which submandibular/ventral oedema was reported (p<0.01); however, as was the case for dysentery (not significant) and haemoglobinuria (p<0.05), which also saw marked increases, this was an infrequently occurring sign. The most striking changes (all highly significant; p<0.001) were observed for anaemia/pallor, weakness and staring coat. All commonly identified in around one quarter to a third of cases during Phase-1, the percentage of cases in which anaemia/pallor was observed more than doubled from under a quarter to over a half of all cases in Phase-2, while weakness and staring coat also almost doubled. In general the number of signs reported per case increased from 3.5 in the first phase to 4.9 in the second phase of the study.

**Table 2:** Frequency with which clinical signs on the DST were observed in the two phases of the study, in decreasing order of relative change in frequency.

Clinical Sign	Phase-1		Phase-2		Relative change
	Percentage of cases in which clinical sign observed [95% CI]				(Ratio)
Submandibular or Ventral Oedema	1.5%	[0.6–2.4]	5.9%	[4.2–7.6]	3.7**
Anaemia or Pallor	22.0%	[19–25.1]	54.6%	[51–58.2]	2.4***
Dysentery	1.4%	[0.5–2.3]	3.3%	[2.1–4.6]	2.3
Haemoglobinuria	3.5%	[2.2–4.9]	7.6%	[5.7–9.5]	2.2*
Weakness	28.2%	[24.9–31.5]	54.9%	[51.3–58.5]	1.9***
Staring coat	33.0%	[29.5–36.4]	60.5%	[57–64]	1.8***
Stunted or Pot Belly	8.4%	[6.4–10.5]	15.5%	[12.9–18.1]	1.8**
Icterus	3.1%	[1.8–4.4]	4.8%	[3.3–6.4]	1.5
Lymph node enlarge	33.5%	[30.1–37]	46.3%	[42.7–49.9]	1.4***
Weight loss	43.8%	[40.1–47.4]	53.5%	[50–57.1]	1.2**
Diarrhoea	28.6%	[25.3–31.9]	32.3%	[28.9–35.6]	1.1
Anorexia or Depression	53.4%	[49.8–57.1]	57.4%	[53.9–61]	1.1
Pyrexia/Fever	43.9%	[40.3–47.5]	47.8%	[44.2–51.4]	1.1
Dyspnoea or Coughing	24.5%	[21.4–27.7]	22.6%	[19.6–25.6]	0.9
Constipation	18.0%	[15.1–20.8]	16.3%	[13.7–19]	0.9
Ataxia or Abnormal Behaviour	6.7%	[4.9–8.6]	6.0%	[4.3–7.7]	0.9
Total signs recorded	2521		3657		
Total number of cases	713		747		
Signs per case	3.5		4.9		

Statistical significance: *p<0.05,

** P<0.01,

*** P<0.001.

**Figure 3:**
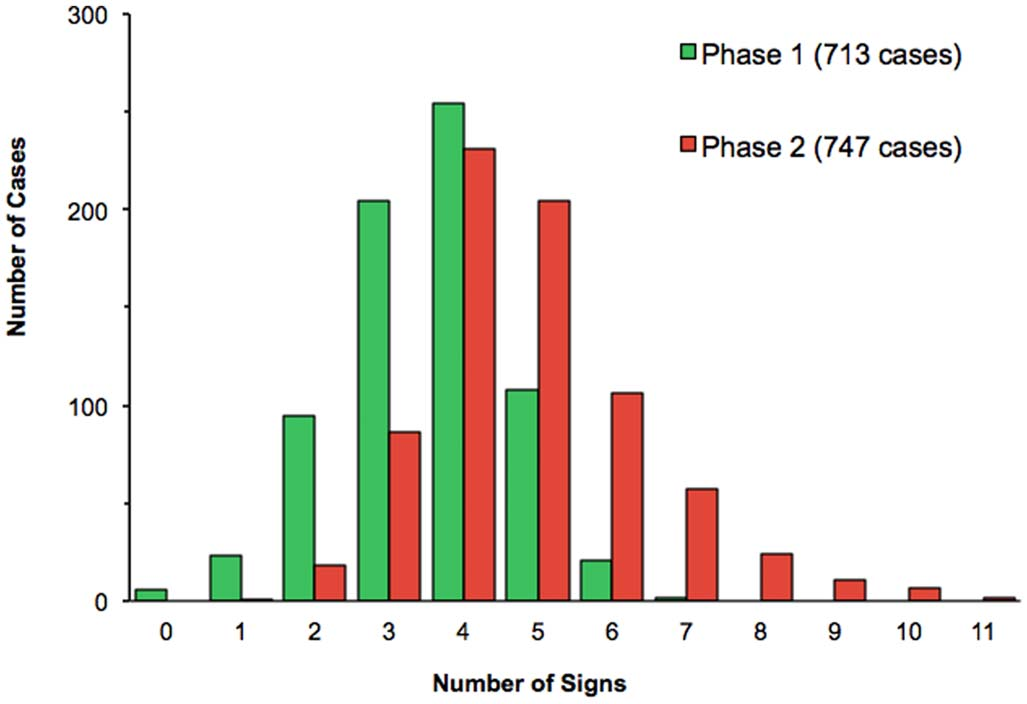
Frequency distribution of numbers of clinical signs recorded for bovine cases by Ugandan veterinary and animal health officers during the two phases of the study. Histogram showing the frequency distribution of number of clinical signs per case observed during each of the two phases of the study, prior to and after the introduction of the diagnostic decision support tool.

The distribution of the number of clinical signs reported per clinical case during each of the two phases is shown in Figure 3, illustrating the structure of the increase in observation and reporting of clinical signs in Phase-2. In only a very few cases during Phase-1 did the participants record six or more signs for a given case; indeed, five signs were recorded in only around 100 cases during the initial phase of the study. In contrast during Phase-2, when the participants were given access to the DST, five signs were recorded in more than 200 cases, and six or more signs were observed in a similar number overall.

**Figure 4:**
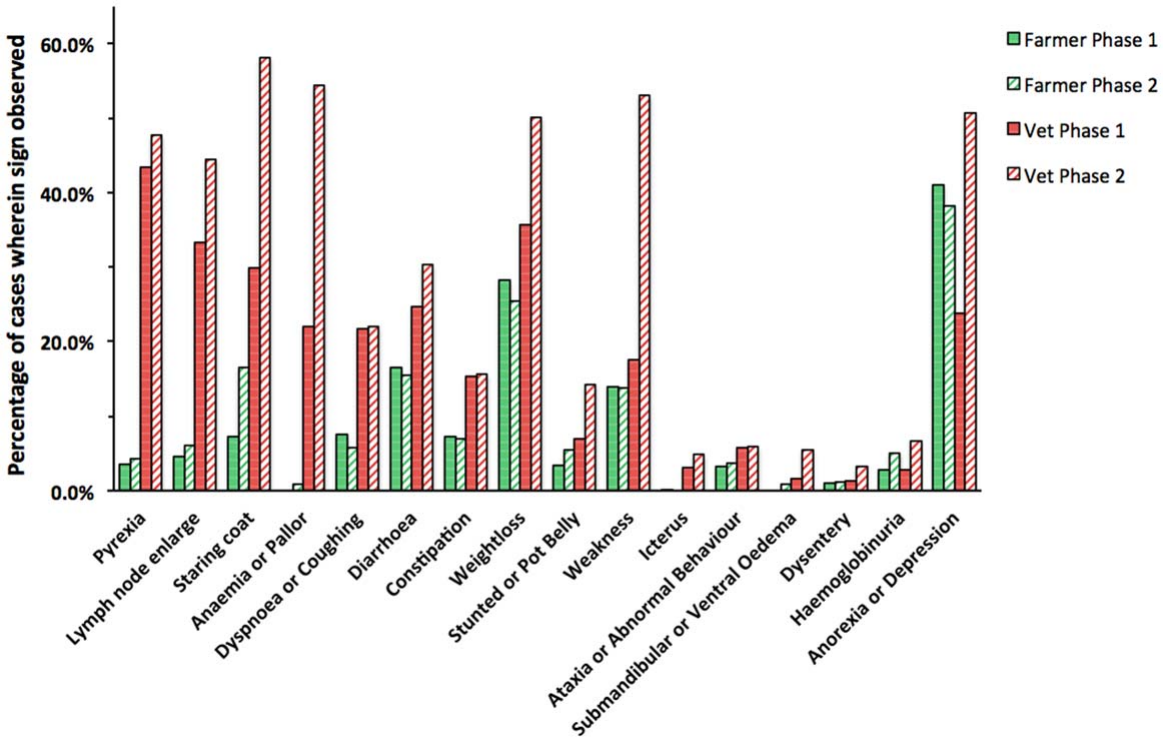
The percentages of bovine cases in which clinical signs were observed by Ugandan farmers and study participants (veterinary and animal health officers) during the two phases of the study. The percentages of clinical cases in which each sign was observed in cattle examined by farmers and study participants during the two phases of the study, ordered by decreasing differences between the two types of observation during Phase-1. (Phase-1, n = 713 cases; Phase-2, n = 747 cases.).

Participants recorded clinical signs as having been observed by themselves, by the farmer or both. The relative frequencies of individual signs identified by each group during Phases 1 and 2, are shown in Figure 4. The overall increase in mean number of signs reported per case was mostly accounted for by an increase in signs observed by participants from 2.9 to 4.7 per case, whereas those observed by farmers changed minimally from 1.4 to 1.5 signs per case. The sign most frequently reported by farmers was anorexia or depression, observed in 39.5% of cases over the two phases, and (in Phase-1) this was the one sign observed more frequently by farmers than participants. Other signs observed by farmers were weight loss, diarrhoea, weakness and staring coat in 26.8%, 16%, 13.8% and 12% of cases respectively in both phases; of these only staring coat showed an appreciable change between the two phases, more than doubling from 7.3% of cases in Phase-1 to 16.5% in Phase-2.

It should be noted that the signs participants were requested to document were not limited to those listed on the DST, and some reported additional signs. These were noted in around 19% of cases, predominantly in Phase-1 of the study. The diagnosis most commonly associated with additional signs was theileriosis (37% of all ‘others’ noted) followed by trypanosomosis (31%), anaplasmosis (22%) and PGE (11.4%). The remaining diagnoses were associated with fewer than 5% of ‘other’ signs noted. The most commonly observed specific signs not listed on the DST were lacrymation (almost 30% of the total), followed by dullness (17%) and nasal discharge (13%). Dehydration, low milk yield and corneal opacity each accounted for around 10% of the total, while a few other specific signs were observed only in a single case.

### Proportional Morbidity in Bovine Cases Examined by Participants

There were four cases in Phase 2 for which participants’ diagnoses were missing and so the final data set available for analysis consisted of 713 and 747 animals from Phase-1 and Phase-2 respectively. These 1460 cases were associated with a total of 1756 participants’ diagnoses; in 291 (19.9%) cases participants noted that two diagnoses were likely, while in 5 cases (all in Phase-1) three possible diagnoses were reported. Where more than one diagnosis was noted, these were weighted equally in the analysis, i.e. each of two diagnoses assigned to a case contributed half, and each of three one third, of the weighting assigned to singleton diagnoses.

**Figure 5:**
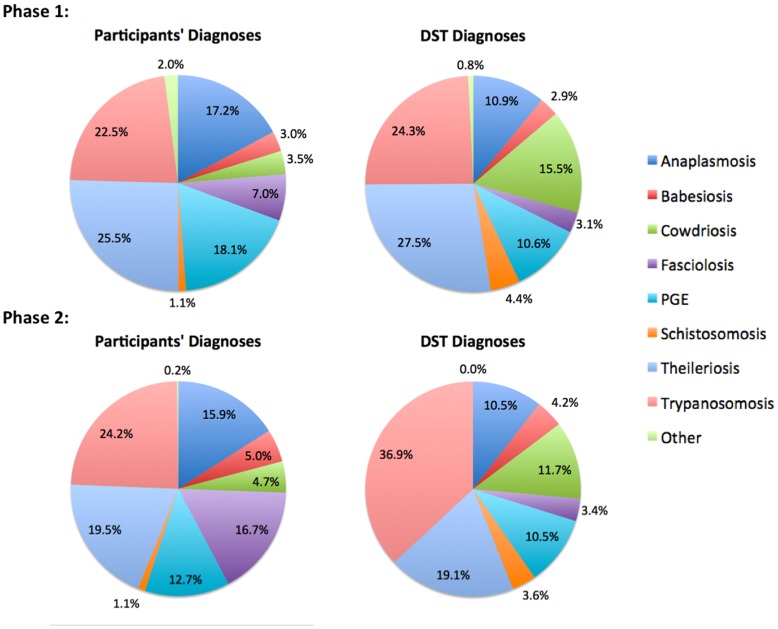
Approximate proportional morbidity in cattle in the eastern region of Uganda based on participating veterinary and animal health officers’ diagnoses and those suggested by the DST. Numbers of individual diagnoses made in each of the two phases of the study are expressed as a proportion of all diagnoses made in that phase. Phase-1 (n = 713 cases) was prior to, and Phase-2 (n = 747 cases) after, the introduction of the diagnostic decision support tool.

The approximate proportional morbidity, i.e. relative frequency, of each of the eight diseases covered by the DST in each of the two study phases is illustrated in Figure 5, based on both participant’s and DST diagnoses. Over the two phases combined, the most common participants' diagnoses were trypanosomosis and theileriosis, representing 23.4% and 22.5% respectively of all diagnoses, followed by anaplasmosis (16.5%), PGE (15.5%) and fasciolosis (12.0%). The proportions of participants’ diagnoses for babesiosis and cowdriosis were fairly low (both less than 5%), with schistosomosis being the least frequently reported diagnosis, accounting for around 1% of all cases. A few diagnoses were made of conditions not included on the DST, these being in Phase-1 contagious bovine pleuropneumonia (5 cases), lumpy skin disease (3 cases), black quarter, mastitis (twice each), metritis, foot and mouth disease and fracture (once each), and in Phase-2 two diagnoses of lumpy skin. Categorised as ‘*other*’, these accounted for only 2.0% of all diagnoses in Phase-1, 0.2% in Phase-2 and 1.1% overall.

The participants’ rates of diagnosis were assessed for departure from the null hypothesis that these were unaffected by the key variables outlined in Table 1 using Chi-squared tests. For neither sex of animal nor participant type was there evidence of difference from the expected disease profiles (p>0.5). However, breed effects were observed; while most tick-borne diseases were consistent with the null hypothesis (i.e. in proportion to the overall number of cases in each breed category) this was not the case for theileriosis, of which levels were significantly higher in crossbred and Friesian cattle and lower in Zebu (p<0.001). Conversely for both PGE (p<0.05) and trypanosomosis (p<0.001) there were significantly higher proportions of cases in Zebu cattle.

There were also interesting departures from the null hypothesis when district was considered. Sironko had significantly higher proportions for the tick-borne diseases anaplasmosis and theileriosis (p<0.001); conversely this district appeared to have significantly lower proportions of trypanosomosis and fasciolosis (p<0.001). The proportion of PGE participants observed was significantly higher in the districts of Iganga and Tororo (p<0.001), for Tororo this elevated level predominantly during Phase-1.

For the final key variable, age group, almost all diseases except cowdriosis and schistosomosis (of which too few cases to test meaningfully for differences) differed significantly (p<0.01) from expectation under the null hypothesis that rate of diagnosis of is unaffected by age (Table 3).

**Table 3:** Breakdown of participants’ diagnoses by age of animal (n = 1460) across the two phases of the study.

Age group	Total	ANA* ^1^	BAB* ^2^	COW	FAS* ^1^	PGE* ^2^	THL* ^2^	TRY* ^2^
0–6 months	**14%**	5%	0%	6%	10%	22%	34%	2%
7–12 months	**26%**	28%	24%	20%	23%	37%	33%	15%
13–24 months	**22%**	26%	41%	29%	17%	19%	17%	26%
>24 months	**38%**	41%	35%	45%	51%	22%	16%	58%

* Differs significantly from the proportions expected under the null hypothesis that rate of diagnosis is unaffected by age: ^1^p<0.01, ^2^p<0.001; schistosomosis not shown as the number of cases (n = 16) was too small to meaningfully test for differences. [ANA = anaplasmosis, BAB = babesiosis, COW = Cowdriosis, FAS = Fasciolosis, PGE = parasitic gastroenteritis, THL = theileriosis, TRY = trypanosomosis.].

### Comparison of the DST with Participants’ Diagnoses

Figure 5 shows the relative frequency of diagnoses suggested by participants and by the DST based on clinical signs they reported during each of the two phases of the study. Across the two phases, the DST suggested diagnoses in proportions broadly similar to those of the participants: namely a predominance of trypanosomosis (30.8% overall), this somewhat higher than for participants’ diagnoses (23.4%), and theileriosis (23.2%); substantial levels of anaplasmosis (10.7%) and PGE (10.6%) albeit these proportions lower than those for participants’ diagnoses (16.5% and 15.5% respectively); and rather lower levels of babesiosis and schistosomosis (both <4%). Contrastingly, the overall proportion of cowdriosis (13.6%) was considerably higher, and that of fasciolosis (3.2%) considerably lower, than those for the participants’ diagnoses (4.1% and 12.0% for these two diseases respectively).

Figure 5 breaks down both the DST and participants’ diagnoses within each of the two phases, and further differences are apparent. Notably, whereas cowdriosis was only the fifth or sixth most common participants’ diagnosis in either phase, representing fewer than 5% of the total, in Phase-1 it was the third most common diagnosis suggested by the DST, accounting for 15.5%. The DST also yielded a significantly higher proportion (P<0.001) of trypanosomosis diagnoses in Phase-2 (36.9%) than it did in Phase-1 (24.3%) or participants did in either phase (23.4%); reductions in DST diagnoses of theileriosis (Phase-1, 27.5%; Phase-2, 19.1%; P<0.001) and cowdriosis (Phase-1, 15.5%, Phase-2, 11.7%; P<0.05) complemented this change. Participants diagnosed fasciolosis more than twice as frequently in Phase-2 (16.7%) as they did in Phase-1 (7.0%), a significant increase (p<0.001), and five-fold more often than did the DST in either phase (3.2% overall); complementary reductions were seen in participants diagnoses of theileriosis (Phase-1, 25.5%, Phase-2, 19.5%; p<0.05) and PGE (Phase-1, 18.1%; Phase-2, 12.7%; p<0.01).

#### Concordance

Concordance (κ) is a measure of agreement corrected for chance between the diagnostic outcomes (be they both presence or absence) of the two diagnostic ‘arms’ of the study, namely participants’ diagnoses and those suggested by the DST. The concordance matrices (κ) shown in Table 4 provide this information for all possible combinations of outcomes over each of the two phases of the study. Arguably of equal interest to the levels of concordance between the DST and the participants for individual diseases as stated on the main diagonal of the κ matrix, are *cross-agreements* between results for different diseases shown by the non-diagonal entries.

**Table 4:** Concordance matrices (κ) of participants’ diagnoses and diagnoses calculated by the DST using clinical signs recorded during the study.

	DST Diagnosis:								
Participant Diagnosis	ANA	BAB	COW	FAS	PGE	SCH	THL	TRY	Other
**Phase-1**									
ANA	**0.586**	−0.029	0.130	−0.055	−0.151	−0.061	−0.114	−0.212	−0.016
BAB	−0.050	**0.807**	−0.029	−0.032	−0.044	−0.037	−0.048	−0.038	−0.013
COW	−0.056	−0.032	**0.322**	−0.034	−0.056	−0.040	−0.066	−0.060	−0.014
FAS	−0.060	−0.037	−0.064	0.176	0.058	0.057	−0.110	0.103	−0.015
PGE	−0.118	−0.052	−0.052	0.102	**0.444**	0.059	−0.238	−0.002	−0.016
SCH	−0.021	−0.016	−0.004	−0.017	−0.021	**0.242**	−0.022	0.001	−0.010
THL	−0.165	−0.049	−0.083	−0.058	−0.162	−0.080	**0.733**	−0.283	0.000
TRY	−0.108	−0.051	−0.097	−0.021	−0.048	0.042	−0.237	**0.487**	−0.016
Other^1^	−0.035	0.034	−0.012	−0.025	−0.035	−0.029	0.005	−0.006	**0.432**
**Phase**-**2**									
ANA	**0.605**	−0.050	0.065	−0.030	−0.128	−0.048	−0.100	−0.178	0.000
BAB	−0.054	**0.772**	−0.049	−0.008	−0.072	−0.016	−0.061	−0.069	0.000
COW	−0.060	−0.047	**0.457**	−0.041	−0.069	−0.043	−0.060	−0.073	0.000
FAS	−0.130	−0.059	−0.113	0.082	**0.221**	0.013	−0.054	0.038	0.000
PGE	−0.078	−0.047	−0.092	0.096	**0.258**	0.117	−0.126	−0.021	0.000
SCH	−0.017	−0.017	−0.007	−0.017	−0.020	0.121	0.000	0.003	0.000
THL	−0.136	−0.069	−0.001	−0.037	−0.093	−0.043	**0.594**	−0.222	0.000
TRY	−0.132	−0.072	−0.104	−0.046	−0.097	−0.011	−0.232	**0.491**	0.000
Other^1^	−0.004	−0.004	0.019	−0.004	−0.004	−0.004	−0.004	0.000	0.000

κ-values for each DST diagnosis in columns are indicated for each participant diagnosis in rows. Main diagonal (mostly boldface numbers) indicating agreement between like diagnoses, other cells indicating possible cross-agreement between differing diagnoses. Boldface numbers indicate at least ‘fair’ agreement (κ ≥0.2); non-bold, positive numbers indicate slight agreement (0.2> κ >0); negative numbers indicate no cross-agreement, i.e. less than that expected by chance.

^1^Other: any participant’s diagnosis other than the eight conditions listed on the DST; a DST diagnosis of ‘other’ resulted when none of its 16 signs was recorded for a case.

2 SCH: schistosomosis. [Further abbreviations as Table 3.].

A complication is that both the human participant and the DST often suggest multiple diagnoses. For example, in Phase-1 of the study, participants recorded a second diagnosis in 120 cases and a third in a further 5 cases, while in Phase-2 they recorded a second diagnosis in 169 cases, but never a third. The DST suggested four diagnoses in 3 of the 1460 cases across the two phases, three diagnoses in 50 cases and two in 171 cases. The approach to calculating κ-values described above takes this into consideration using weighting values dependant upon the number of diagnoses per case.

Table 5 summarises the information from the main diagonals of the Phase-1 and Phase-2 concordance matrices (Table 4), together with the number of cases on which each diagnosis was based and provides the same information for the two phases combined. Concordance between outcomes of participants’ diagnoses and the DSC differed markedly among diseases, and to a lesser extent between the first and second phases. Overall across both phases, the concordance of the DST with the participants was around 50% for all 1460 cases (κ = 0.486). The level of concordance was actually higher for cases seen in Phase-1 (κ = 0.519) than for those seen in Phase-2 (κ = 0.452) when the participants had the DST to hand. The individual disease having the greatest agreement was babesiosis (κ = 0.786), with similar levels in both phases of the study. Anaplasmosis, theileriosis and trypanosomosis had overall agreement levels of 0.595, 0.672 and 0.491 respectively, again with similar levels across phases, albeit theileriosis having slightly poorer agreement in Phase-2. There was least agreement by far over fasciolosis, particularly during the second phase of the study (κ = 0.082) when a significant overall increase in the proportion of cases participants reported with this diagnosis was unmatched by the number of times this diagnosis was suggested by the DST (Figure 5).

**Table 5:** Concordance (κ) of outcomes for each of the eight diagnoses suggested by the DST with those made by participating veterinary and animal health officers.

Concordance (κ) of DST with Participants’ Diagnoses(n cases with participant’s diagnosis)			
Participant’s Diagnosis:	Phase-1	Phase-2	Both Phases
All diseases	0.519(713)	0.452(747)	0.486(1460)
Babesiosis	0.807(21.5)	0.772(37)	0.786(58.5)
Theileriosis	0.733(182)	0.594(146)	0.672(328)
Anaplasmosis	0.586(122.5)	0.605(119)	0.595(241.5)
Trypanosomosis	0.487(160.7)	0.491(180.5)	0.491(341.2)
Other	0.432(14.5)	0.000(1.5)	0.406(16)
Cowdriosis	0.322(25)	0.457(35)	0.385(60)
PGE^1^	0.444(129.2)	0.258(95)	0.357(224.2)
Schistosomosis	0.242(8)	0.121(8)	0.185(16)
Fasciolosis	0.176(49.7)	0.082(125)	0.113(174.7)

NB the DST was available to participants during Phase-2 but not Phase-1.

^1^As table 3.

In interpreting Tables 4 and 5 it is important to consider that the concordance (κ) values indicate that portion of agreement not accounted for by chance; hence, most of the non-diagonal cells in Table 5 have trivially small or negative values, indicating no agreement. In striking contrast values in most of the diagonal cells indicate a fair to substantial level of agreement (0.807≥ κ ≥0.242), with the notable exceptions of fasciolosis in Phase-1 (κ = 0.176) and Phase-2 (κ = 0.082) and schistosomosis in Phase-2 (κ = 0.121) for which agreement was slight, or poor. A few further cells in the concordance matrices also have non-trivial values. For instance in Phase-1, while the DST recorded moderate concordance with participants for anaplasmosis (κ = 0.586), it also showed slight cross-agreement for that disease with participants’ results for cowdriosis (κ = 0.130). Similarly in Phase-1, the (slight) agreement of the DST with participants over fasciolosis was almost equalled by a slight cross-agreement of participants’ fasciolosis status with the DST’s on trypanosomosis (κ = 0.103); and the moderate level of concordance (κ = 0.444) of the DST with participants over PGE was accompanied by a slight cross-agreement with fasciolosis (κ = 0.102).

Clinical signs were reported as having been observed by either the farmer or the veterinary participants themselves, and DST diagnoses could be calculated using each of these sign sets or the full, combined sign set as was used in Tables 2, 3, 4, 5 and Figure 5. A further possibility is a combined set including all signs observed by veterinary participants and a subset of those observed by farmers restricted to anorexia/depression, ataxia/abnormal behaviour, constipation, diarrhoea, dyspnoea/coughing, staring coat, weakness and weight loss; all of significance for the clinical history and though readily detected, not necessarily apparent during a brief veterinary examination. Using only signs observed by farmers in Phase-1 to calculate DST diagnoses yielded a lower overall κ-value in the ‘fair’ category of just 0.332, compared to the ‘moderate’ value of 0.519 obtained using the full sign set. Phase-1 DST diagnoses calculated using signs observed by veterinary participants alone gave a slightly higher overall κ-value of 0.541, while complementing these signs with the restricted subset of farmers’ signs gave an overall κ-value of 0.510, not appreciably different from that using the full sign set.

Other analyses using DST diagnoses derived from the full sign set in Phase-1 examined the effect of either including only one participant’s diagnosis for each case (the first where more than one was recorded), which resulted in slightly better overall concordance (κ = 0.567), or restricting cases to those where both the DSC and the participant made unique diagnoses (n = 499), which led to higher still Phase-1 concordance (κ = 0.641).

#### Misclassification

Misclassification matrices were scaled to show either, for cases in which the participants diagnosed a particular condition (columns), the proportions of each diagnosis (rows) suggested by the DST (M*_v_*, Table 6a) or, for cases in which the DSC suggested a particular condition (columns), the proportions of each diagnosis (rows) made by participants (M*_c_*, Table 6b). These misclassification matrices enable identification of sources of discrepancy between the two diagnostic arms of the study. For instance, while cowdriosis scores highly (0.980) on the main diagonal of the Phase-1 M*_v_* matrix (Table 6a), indicating that where participants diagnosed this condition the DST suggested likewise in almost every case, the corresponding Phase-1 M*_c_* matrix (Table 6b) shows that only a modest proportion (0.221) of cases suggested as cowdriosis by the DST were diagnosed as such by participants; they also apportioned these cases among anaplasmosis (0.286), theileriosis (0.166), PGE (0.134) and trypanosomosis (0.128). This explains the 12% discrepancy between Phase-1 proportional morbidity (Figure 5) for cowdriosis derived from participants’ diagnoses (3.5%) and the DST suggestions (15.5%), reassures us that virtually all of the 3.5% comprises a part of the 15.5% (there might otherwise have been no commonality between the two) and explains how the 12% difference distributes among participants’ diagnoses.

**Table 6:** Misclassification matrices, (a) M*_v_* and (b) M*_c_*, of participants’ diagnoses and diagnoses calculated by the DST using clinical signs recorded during the study.

(a) M*_v_*		Participant Diagnosis:								
	DST Diagnosis	ANA	BAB	COW	FAS	PGE	SCH	THL	TRY	Other
**Phase-1**	ANA	**0.524**	0.000	0.000	0.039	0.027	0.000	0.009	0.040	0.000
	BAB	0.012	**0.791**	0.000	0.003	0.000	0.000	0.003	0.001	0.069
	COW	**0.259**	0.070	**0.980**	0.062	**0.115**	**0.125**	**0.101**	0.088	**0.103**
	FAS	0.000	0.000	0.000	**0.153**	0.088	0.000	0.000	0.020	0.000
	PGE	0.000	0.012	0.000	**0.173**	**0.410**	0.000	0.008	0.076	0.000
	SCH	0.008	0.000	0.000	0.087	0.077	**0.625**	0.000	0.067	0.000
	THL	**0.159**	0.047	0.000	0.034	0.041	0.000	**0.835**	0.077	**0.310**
	TRY	0.039	0.081	0.020	**0.450**	**0.241**	**0.250**	0.036	**0.631**	**0.207**
	Other	0.000	0.000	0.000	0.000	0.000	0.000	0.008	0.000	**0.310**
**Phase-2**	ANA	**0.543**	0.027	0.014	0.013	0.042	0.016	0.015	0.024	0.000
	BAB	0.013	**0.725**	0.000	0.008	0.013	0.000	0.003	0.003	0.000
	COW	**0.166**	0.041	**0.864**	0.035	0.039	0.078	**0.116**	0.052	**0.667**
	FAS	0.017	0.027	0.000	0.080	0.091	0.000	0.013	0.009	0.000
	PGE	0.013	0.000	0.000	**0.262**	**0.314**	0.000	0.044	0.046	0.000
	SCH	0.008	0.023	0.000	0.043	**0.107**	**0.297**	0.012	0.030	0.000
	THL	**0.100**	0.054	0.050	**0.144**	0.058	**0.188**	**0.666**	0.028	0.000
	TRY	**0.140**	**0.104**	0.071	**0.415**	**0.335**	**0.422**	**0.131**	**0.809**	**0.333**
	Other	0.000	0.000	0.000	0.000	0.000	0.000	0.000	0.000	0.000
**(b)** M**_c_**		**DST Diagnosis:**								
	**Participant Diagnosis**	**ANA**	**BAB**	**COW**	**FAS**	**PGE**	**SCH**	**THL**	**TRY**	**Other**
**Phase-1**	ANA	**0.825**	0.074	**0.286**	0.000	0.000	0.032	0.099	0.027	0.000
	BAB	0.000	**0.836**	0.014	0.000	0.003	0.000	0.005	0.010	0.000
	COW	0.000	0.000	**0.221**	0.000	0.000	0.000	0.000	0.003	0.000
	FAS	0.025	0.008	0.028	**0.343**	**0.114**	**0.139**	0.008	**0.129**	0.000
	PGE	0.045	0.000	**0.134**	**0.513**	**0.702**	**0.322**	0.027	**0.180**	0.000
	SCH	0.000	0.000	0.009	0.000	0.000	**0.161**	0.000	0.012	0.000
	THL	0.021	0.025	**0.166**	0.000	0.020	0.000	**0.774**	0.037	**0.250**
	TRY	0.084	0.008	**0.128**	**0.143**	**0.161**	**0.346**	0.063	**0.585**	0.000
	Other	0.000	0.049	0.014	0.000	0.000	0.000	0.023	0.017	**0.750**
**Phase-2**	ANA	**0.825**	0.047	**0.225**	0.079	0.019	0.037	0.083	0.060	NA^1^
	BAB	0.013	**0.850**	0.017	0.040	0.000	0.031	0.014	0.014	NA
	COW	0.006	0.000	**0.345**	0.000	0.000	0.000	0.012	0.009	NA
	FAS	0.020	0.032	0.049	**0.397**	**0.416**	**0.201**	**0.126**	**0.188**	NA
	PGE	0.051	0.040	0.043	**0.344**	**0.379**	**0.377**	0.039	**0.115**	NA
	SCH	0.002	0.000	0.007	0.000	0.000	0.088	0.011	0.012	NA
	THL	0.028	0.016	**0.194**	0.076	0.081	0.065	**0.680**	0.069	NA
	TRY	0.056	0.016	**0.107**	0.063	**0.105**	**0.202**	0.035	**0.529**	NA
	Other	0.000	0.000	0.011	0.000	0.000	0.000	0.000	0.002	NA

In (a), **M** ***_v _***, proportions of each participant diagnosis are shown for each DST diagnosis in columns summating to 1. In (b), **M** ***_c _***, proportions of each DST diagnosis are shown for each participant diagnosis in columns summating to 1.

Leading diagonal indicating agreement, other cells indicating disagreement.

^1^NA: not applicable. [Other abbreviations as Table 4.].

Other differences between participants’ and DST diagnoses manifest in Figure 5 as differences in proportional morbidity may be similarly explained using Table 6. Compared with participants, the DST apparently under-diagnosed anaplasmosis markedly in both phases; only around half (Phase-1, 0.524; Phase-2, 0.543) of cases diagnosed as anaplasmosis by participants were suggested as such by the DST, the discrepancy distributed among cowdriosis (Phase-1, 0.259; Phase-2, 0.166), theileriosis (Phase-1, 0.159; Phase-2, 0.100) trypanosomosis (Phase-1, 0.039; Phase-2, 0.140) and to a much lesser extent other conditions (Table 6a). Where the DST suggested anaplasmosis, participants agreed with a high proportion (0.825) of these diagnoses in both phases (Table 6b). With PGE, the situation was less clear-cut; while proportional morbidity was higher for participants’ diagnoses (Phase-1, 18.1%; Phase-2, 12.7%) than DST suggestions (Phase-1, 10.6%; Phase-2, 10.5%), for both diagnostic arms of the study Tables 6a and b reveal misdiagnoses distributed among a number of conditions, reflecting the relatively low concordance (κ = 0.357) for this condition across both phases (Table 5). Similarly for fasciolosis, which had the lowest concordances of all in both phases and overall (κ = 0.113, Table 5), Table 6a reveals that participants’ diagnoses of this condition in either phase are mostly apportioned by the DST to trypanosomosis (Phase-1, 0.450; Phase-2, 0.415), PGE (Phase-1, 0.173; Phase-2, 0.262), theileriosis (Phase-2, 0.144) or fasciolosis itself (Phase-1, 0.153), and Table 6b shows that DST-suggested diagnoses of fasciolosis are likewise mostly apportioned by participants among PGE (Phase-1, 0.513; Phase-2, 0.344), fasciolosis (Phase-1, 0.343; Phase-2, 0.397) or trypanosomosis (Phase-1, 0.143). Finally, Table 6b shows that in Phase-2, the additional proportional morbidity (Figure 5) attributed to trypanosomosis by DST diagnoses (36.9%) compared with that attributed by participants’ diagnoses (24.2%) was explained by cases apportioned by participants among fasciolosis (0.188) and PGE (0.115); and Table 6a shows correspondingly that where participants diagnosed trypanosomosis in Phase-2, a high proportion of DST suggested diagnoses agreed (0.809).

## Discussion

This study evaluated the effectiveness of a decision support tool as a diagnostic aid under field conditions in Uganda by observing whether its introduction to veterinary and animal health officers undertaking primary animal health care would affect their clinical practice in terms of observation of clinical signs and arrival at specific diagnoses. Fifteen participants including District Veterinary Officers, Veterinary Officers and Animal Health Officers from five districts in Uganda provided information on 1442 bovine clinical cases for which they reported a total of 6152 clinical signs. During an initial phase, participants reported clinical signs and diagnoses for 713 cases based on their usual practice, whereas in a subsequent phase clinical signs and diagnoses were reported for a further 751 cases investigated using a simple, low cost decision support tool for differentiation amongst eight common conditions. While livestock owners’ perceptions of cattle diseases and their treatments have been investigated previously [1], [24], [25], this appears to be the first study of diagnoses by veterinary staff or animal health assistants under field conditions in the Lake Victoria Basin.

The composition of the case data sets remained broadly similar between the two phases in terms of a number of variables, namely animal age group, gender and breed, administrative district and participant type. No instructions were given to the participants in terms of case selection so we can assume that this breakdown is broadly reflective of the cattle for which they were receiving requests to carry out diagnosis. Of interest is the fact that clinical examinations were around three times as likely to involve animals in the over 24-month category than they were very young cattle (0–6 months) and that around twice as many female cattle were presented as were male, perhaps reflecting the higher perceived value of adult females in terms of reproductive potential and/or milk production; similar bias towards older animals and females was observed by Van den Bossche et al. [26] in relation to farmer use of trypanocidal drugs in cattle in Zambia.

### Clinical Signs Recorded by Participants

A striking feature of this study was the increase in the average number of clinical signs per case observed following the introduction of the DST, 4.9 in Phase-2 compared with 3.5 in Phase-1. Almost 28% of Phase-2 cases showed six or more signs compared to just 3% of cases in Phase-1 (Figure 3). The individual signs contributing most to this increase were anaemia/pallor, weakness and staring coat, which all but doubled in number in Phase-2 to be seen in over half of all cases. However, while the overall number of signs reported increased in Phase-2, this increase was limited to signs listed on the DST; some other signs reported during Phase-1 but not on the DST, such as lacrymation, dullness and nasal discharge were no longer reported after its introduction. The interpretation of this may be that while the DST encourages clinical examination and recording of signs observed, this effect is limited to those signs listed on it. Finally, while dullness might be considered equivalent to anorexia/depression as listed on the DST, addition of anorexia/depression as a sign to 14 Phase-1 cases that lacked it but nevertheless reported dullness did not substantively affect the results.

Also of interest was the ability of farmers to identify clinical signs, some of which might not be manifest at the time of the veterinary staff visit, but would be reported as clinical history. Work in neighbouring Kenya showed that cattle keepers in production systems with similar disease challenge identified a number of clinical signs associated with bovine trypanosomiasis including staring coat, inappetence, weight loss, eating soil (pica), nasal discharge, weakness, coughing, constipation, salivation, dullness, lameness, diarrhoea, reluctance to drink, swollen lymph nodes and tooth grinding [1]. In the present work, farmers were shown to identify some signs more frequently than others, and in the case of anorexia or depression more frequently than veterinary staff, at least prior to the introduction of the DST (Figure 3). It can be envisaged that the DST might be helpful to farmers in diagnosing endemic disease in their cattle, and the results obtained here support earlier work in confirming they are able to identify at least some clinical signs, but also suggest that they would benefit from additional training to facilitate this.

### Proportional Morbidity

Given that the participants’ diagnoses were available for both the first and second phases of the study, we initially used these to characterise the disease status of the animals examined as a measure of proportional morbidity in the population under the clinical care of the participants. The eight diseases covered by the DST included over 98% of the putative diagnoses made by the participants for cases they attended throughout the study period, and hence the DST diagnoses may also provide an approximate measure of proportional morbidity in these districts of Uganda. In around 20% of cases the participants noted that more than one diagnosis was likely; primarily this was a second possible diagnosis, though in Phase-1 there were 5 cases where three possible diagnoses were reported.

The conditions most commonly diagnosed by participants in both phases were three vector-borne diseases: trypanosomosis, theileriosis, and anaplasmosis, and two helminthiasies: parasitic gastroenteritis (PGE) and fasciolosis; babesiosis, cowdriosis and schistosomosis were far less frequently diagnosed. While trypanosomosis remained the most common diagnosis through both phases, representing around a quarter of all diagnoses, theileriosis and PGE were diagnosed significantly less commonly in Phase-2, effectively being replaced by a significant increase in fasciolosis, for which proportional morbidity doubled. These diagnoses were consistent with endemic diseases reported by other studies of cattle in the region [1], [24], [25], and are reflected in the range of drugs stocked by agro-veterinary shops in the region and their rates of sale [27]. No significant transboundary disease epidemics occurred during the study period.

Independent measures of occurrence of diseases within the target population, i.e. cattle under the primary animal health care of the veterinary and animal health officers participating in the study, would be of interest in evaluating the impact and performance of the DST, but there are few formal studies and reliable contemporary prevalence and incidence data are not available. Hence, while possible confounding and various uncontrolled sources of bias suggest that care must be taken not to over-interpret these results, it is interesting to comment on the observed rates of diagnosis (proportional morbidity), and compare these to values expected under the null hypothesis that rates were unaffected by the key variables outlined in Table 1. Specifically, there appeared to be predisposition towards diagnosis of theileriosis in Friesian and crossbred cattle, as compared with Zebu, whereas Ankole appeared to have greater likelihood of diagnosis of trypanosomosis than other breeds, observations consistent with known breed susceptibilities [28]. Similarly, some district level effects were observed, such as lower proportional morbidity due to trypanosomosis and fasciolosis in Sironko District, consistent with its higher elevation on the slopes of Mount Elgon, and that most diagnoses of schistosomosis in were made in Kayunga District, but it was difficult to draw any firm conclusions from these effects. The results reported here are based on data collected over two specific periods in a single year across the areas under consideration. To obtain insight into seasonal and annual variation it would be necessary to utilise the DST as an on-going diagnosis/monitoring tool.

Age effects on proportional morbidity were possibly of greater interest (Table 3). In this area of Uganda, there appears to be clear evidence that diagnosis of PGE is primarily associated with young animals while the opposite appears to be the case with trypanosomosis (significantly fewer cases in the two age classes of animals under one year and significantly more in those cattle of two years or older). However, it is arguably the age distributions for cases diagnosed with tick-borne diseases that are most interesting. For three of these (anaplasmosis, babesiosis and cowdriosis) there is evidence that the presence of disease in young animals (less than 6 months old) was significantly lower than would be expected based purely on proportions of cattle in each age group, consistent with the concept of inverse age immunity for these diseases [29]. However, this was clearly not the case for theileriosis for which the proportion with the disease was significantly higher than expected in younger and significantly lower in older animals, consistent with observations by David Bruce et al. almost 100 years earlier [18].

### Comparison of the DST with Participants’ Diagnoses

Previous attempts at evaluating expert systems for animal disease diagnosis have used selected test cases [5], [30], whereas the present study was based on naturally occurring disease. Hence, one challenge in evaluating the performance of the DST was not having an independent assessment as to which disease or diseases were truly present in each of the cases. Unfortunately provision of definitive diagnostic capability through laboratory investigations would have been prohibitively costly and beyond the scope of this study. Even if that had been feasible, detailed diagnostic investigations in remote African rural settings might have influenced interactions between farmers and veterinary participants and introduced significant bias to the study. In the absence of such a ‘gold standard’ to evaluate whether the tool achieved the ‘correct’ diagnosis we made no assumption that the human participant (veterinary or animal health officer) made the correct diagnosis and but simply assessed how often the suggested diagnosis of the DST was in agreement (Tables 4 and 5). Moreover, we note that comparisons of the two types of diagnosis differ between the two phases; Phase-1 comparison was between the DST’s rendition of clinical signs reported by participants not yet introduced to it and diagnoses based on their customary clinical practice, whereas in Phase-2 the comparison was between the DST’s rendition of reported clinical signs and diagnoses suggested by participants using the DST as an aid to their clinical judgement.

A further complication is that both human observers and the DST may suggest multiple diagnoses. The DST may allocate the same score to multiple diseases for a given combination of clinical signs; of the 65,536 (2^16^) possible sign combinations, 18,352 (28.0%) yield more than one diagnosis. Indeed, the concordance values reported here must be interpreted taking into consideration that neither the participants’ nor the DST diagnoses returned perfect scores when compared with themselves (participants Phase-1, overall κ = 0.894; DST Phase-1, overall κ = 0.886). Within either diagnostic arm of the study, cases with more than one diagnosis may be regarded as having internal disagreement. In terms of individual diseases, this ‘*self-concordance*’ of the participants’ diagnoses was in the ‘almost perfect’ category (κ >0.8) for all but fasciolosis (κ = 0.770), although no particular ‘cross-agreement’ was evident. Likewise, self-concordance of the DST was in the almost perfect category (κ >0.8) for all diseases but fasciolosis (κ = 0.634) and schistosomosis (κ = 0.635), these two having a weak mutual cross-agreement (κ = 0.072).

Concordance of the DST with participants’ diagnoses is best examined in detail for Phase-1, when the DST had not yet been introduced to the participants (Table 4). For example, for the DST’s result with regard to cowdriosis, in addition to a ‘fair’ level of agreement (κ = 0.322) with veterinary participants’ results for the same condition, the DST also showed slight cross-agreement (κ = 0.130) with a participant’s result for anaplasmosis. This can be contrasted with the sixth column in the Phase-1 concordance matrix (Table 4), which shows the level of (cross-)agreement of participants’ results for each disease with the DST’s result for schistosomosis. While this indicates that the DST was in fair agreement (κ = 0.242) with the participants with regard to presence or absence of schistosomosis, the DST also cross-agreed with the participants’ results for fasciolosis, PGE and trypanosomosis. Despite the individual values being small, schistosomosis accrued by far the highest level of cross agreements.

Additional concordance matrices (not shown) were derived using a number of alternative approaches to the data, for instance including or not including various combinations of clinical signs observed by either farmers or participating veterinary staff in calculating the DST diagnoses, or using only the first of multiple participants’ diagnoses for a particular case. Generally, clinical signs observed by farmers resulted in DST diagnoses with poorer agreement with participants’ diagnoses, whereas perhaps unsurprisingly using only a single participants’ diagnosis for each case improved concordance with the DSC, probably on account of ‘perfect’ self-concordance (κ = 1.000) achieved by having unique participants’ diagnoses. The highest Phase-1 concordance (κ = 0.641) was obtained using DST diagnoses derived from the full sign set and restricting cases to those where both the DSC and participants made unique diagnoses, again perhaps unsurprising given perfect self-concordance in both arms of the study.

Both concordance and misclassification were useful in assessing the performance of the DST in relation to participants’ diagnoses. While concordance is the proportion of agreement corrected for chance taking into consideration both ‘positive’ and ‘negative’ agreements, i.e. where both participants and the DST agree that the diagnosis either is or is not a particular condition, misclassification more simply represents positive agreements uncorrected for chance. Hence while concordance is a better measure of whether there was agreement between the two diagnostic arms of the study, it gives only limited indication of where disagreements lie. The misclassification matrices are less informative about agreement between the two but enable, for each (‘positive’) diagnosis made by one study arm, assessment of what proportion of (‘positive’) diagnoses were allocated to that diagnosis or alternative diagnoses by the other arm. Misclassification matrices were particularly useful in establishing how observed differences in proportional mortality between the two arms could be explained in terms of the breakdown of individual disease diagnoses.

Clearly because of the complication of multiple diagnoses these results could not easily be reduced to simple dichotomous outcomes (matched or not) from which specificity and sensitivity scores could be estimated on the basis of one or other diagnosis as ‘gold standard’. The authors feel that the full concordance and misclassification matrices shown in Tables 4 and 6 are more informative and suggest these as a useful approach to the evaluation of this type of low-cost diagnostic decision support tool.

Finally, in addition to diagnoses, participants in this study also reported on the outcome of clinical cases, these being described as ‘good’ (as opposed to ‘poor’ or ‘fair’) in 88.4% of cases over both phases of the study although outcomes assessed by the participating clinician and not by an independent observer cannot be regarded as unbiased. Indeed, this success rate may seem rather optimistic in the face of such severe endemic disease challenge, but it was nevertheless interesting to note that there was a small but statistically significant increase (p<0.001) in the percentage of cases with a good outcome from 85.5% in Phase-1 to 92.1% in Phase-2; further studies would be required to assess whether this increase on the introduction of the DST held with unbiased observation.

### Conclusion

In this study is was possible to compare the diagnostic performance of veterinary and animal health officers undertaking primary animal health care in Uganda before and after the introduction of a decision support tool and investigate changes in clinical practice in terms of observation of clinical signs and arrival at specific diagnoses. The decision support tool was shown to be highly relevant to the study area in that it covered the vast majority of diagnoses made before or after its introduction. The diagnoses suggested by the decision support tool were broadly consistent with those made by veterinary and animal health officers, but there was variation across diseases with some individual diagnoses (fasciolosis, schistosomosis and PGE) showing less consistency. Concordance and misclassification matrices were useful in establishing levels of agreement and details of where differences lay. Importantly, the introduction of the diagnostic decision support tool led to a significant increase in the number of clinical signs recorded by the participants, suggesting this as an key beneficial consequence of its use over and above any improvement of the diagnosis made using a given sign set. In this regard one of the benefits of the DST can be regarded somewhat similar to how use of diagnostic "checklists" in human hospitals can increase efficiency and reduce missed clinical signs or mistaken diagnoses [31].
